# Re-imagining students healing, power and belonging through eco-therapy based peer-led action

**DOI:** 10.1371/journal.pmen.0000663

**Published:** 2026-07-23

**Authors:** Peris Mutena

**Affiliations:** Department of Psychology, Counselling and Educational Foundations, Egerton University, Nakuru, Kenya; PLOS: Public Library of Science, UNITED KINGDOM OF GREAT BRITAIN AND NORTHERN IRELAND

## The crisis of disconnection and a return to our roots: Nature and movement

University students are increasingly experiencing significant mental health challenges worldwide. Research on university student mental health shows that psychological and academic pressures intersect with a combination of socio-economic and structural factors. As an author based in Kenya, I am writing with the Kenyan context at the forefront of my mind, although many of the points discussed in the Essay can apply globally. In the Kenyan context for instance, financial stress is consistently identified as a major contributor to student distress, alongside academic workload, family pressures and social adjustment challenges [1]. The transition into university life further intensifies vulnerability, with first-year students experiencing significant adjustment stress as they adapt to new academic, social and financial demands [2]. A large multi-country study of university students found that psychological distress levels are notably high across institutions of further education, with over 60% of students reporting moderate to severe stress, and approximately 35–50% experiencing clinically significant anxiety symptoms depending on the measurement scale used. The study further showed that anxiety levels were consistently elevated in students facing financial difficulties, academic overload and poor social support systems. These findings confirm that university students represent a high-risk group for mental health disorders, particularly anxiety and stress-related conditions, with prevalence rates significantly higher than in the general population [2].

Together, these intersecting pressures contribute to a deeper crisis of disconnection from self, community and natural environment. Within fast-paced, performance-driven environments people are often expected to remain productive and resilient without adequate opportunities to pause, reflect and reconnect with their bodies or cultivate meaningful relationships. As stress accumulates, students may become increasingly disconnected from their emotional needs, supportive social networks and the environments that promote wellbeing.

As these pressures continue to accumulate, students may adopt coping strategies that provide short-term relief but do not necessarily address the underlying sources of distress. Evidence shows that students experiencing prolonged stress often rely on maladaptive coping mechanisms such as social withdrawal, avoidance and substance use [1]. Despite the significant personal burden of mental health challenges, many students do not seek formal support due to stigma, limited mental health literacy and fear of negative social judgement [3]. This creates a gap between the increasing need for mental health support and the accessibility of existing systems.

At the institutional level, studies show that university mental health support systems in many low- and middle-income countries are under-resourced and reactive; limiting early detection and intervention for students in distress [4]. Overall, these findings highlight that university student mental health is shaped by intersecting academic, economic, social, and systemic factors; with financial insecurity and weak support structures playing a central role in worsening psychological outcomes.

Notably, students frequently turn to informal support networks before engaging with professional services. Research indicates that up to 75% of students experiencing mental health difficulties first disclose their struggles to a peer [5], highlighting the central role of peer relationships in early support and intervention.

Peer support interventions have demonstrated positive outcomes, including improvements in recovery, social functioning and quality of life, highlighting their potential as complementary, culturally responsive and scalable approaches to student mental health care [6]. Peer support offers meaningful relationships that provide emotional, informational and practical resources, enabling individuals to cope with challenges through connection and belonging [7]. Extending this perspective, empowerment theory emphasizes that supportive environments not only provide assistance but also cultivate agency, self- efficacy and the capacity of individuals and communities to participate actively in creating change [8]. Mutual Aid Theory highlights the importance of reciprocal relationships, suggesting that collective care and shared responsibility are fundamental human processes that support resilience and social wellbeing [9]. Together, these theories position peer support as more than a therapeutic technique, it becomes a relational process through which individuals reconnect with others, develop collective strength and become empowered participants in their own healing and community transformation.

Traditional peer support models in higher education have largely focused on conversational approaches, where students provide empathy, advice and emotional validation through dialogue. While these interactions remain valuable, there is a need to expand peer support beyond primarily talk-based approaches toward more embodied and experiential forms of connection that address the broader crisis of disconnection.

An embodied eco- therapeutic approach frames peer support as a structured, co-regulatory process, that incorporates the body, relationships and the natural environment. Eco-therapy is a nature-based approach to wellbeing that recognizes the relationship between individual mental wellness, peer community support and the natural environment. It involves intentional activities such as mindfulness, mindful walking, outdoor reflection, gardening, circle sharing, sensory engagement, and other forms of interaction with nature that support psychological, emotional, and physical wellbeing. Eco-therapy supports mental health through mechanisms such as restoration of attention, reduction of cognitive overload, increased present-moment awareness and strengthening of connection with the environment [10,11]. Furthermore, eco-therapeutic processes such as yoga and mindfulness based approaches have shown to improve emotional regulation and reduce symptoms associated with stress and anxiety [12,13]. Within peer-led framework, eco-therapy therefore extends peer support beyond emotional disclosure into shared embodied practices that promote resilience, social connection and psychological wellbeing. In this model, peers are not only emotional listeners but also co-participants in regulating stress responses through the body and then environment, thereby reducing reliance on purely verbal processing. This approach provides healing spaces for the university student community aligning with evidence that peer support can be enhanced when it is structured, owned by students through co-creation and embedded within everyday student life [5]. Through the Indigenous knowledge systems approach where most African communities traditionally healed through communal gatherings, storytelling, connection to land and collective care [14], this shift reframes support from a reactive, talk based system into a preventative, embodied and relational practice that integrates the body, environment and peer connection as active mechanisms of healing through efficient training and guidance by professionals in nature based interventions such as mindfulness and sensory engagement to ensure exercises are undertaken safely and can also be undertaken in a students’ classroom environment.

This Essay advances a central argument eco-therapy as a framework for addressing student mental health through peer community connectedness and environmental stewardship in institutions of higher learning. It argues that student distress is, in part, a crisis of disconnection from nature, community, and self and that eco-therapy offers a culturally grounded response by restoring these connections. In doing so, the essay reimagines peer support beyond traditional talk-based approaches, positioning it as an embodied, nature-based, and relational practice through which students become active co-creators of wellbeing, belonging and environmental care.

This framework responds directly to the crisis of disconnection by restoring relationships with the environment, people around you and the present body; In re-anchoring in the present moment. It does this through structured nature based activities such as guided outdoor reflection, mindful walking, gardening and sensory engagement with natural settings. These practices reduce rumination, stress and anxiety by shifting attention away from internal cognitive overload toward embodied, present-moment awareness and environmental attunement [15]. In addition, eco-therapy strengthens emotional regulation by fostering reciprocal relationship with nature, where individuals not only receive calming and restorative effects from natural environments but also experience a sense of belonging and interaction with it, which supports meaning-making and psychological recovery [15,16]. Furthermore, young people are empowered; not just as participants but as co-creators of this framework through student selecting discussion themes, sharing personal reflections in facilitated peer support and contributing to designing mindfulness activities.

## Eco-therapy as a form of Epistemic Repair

Eco-therapy is anchored in the Indigenous Knowledge Systems (IKS) understanding humans as an interconnected ecological community, where relationships with land, plants and other living beings contribute to individual and collective wellbeing. [14], by suggesting ‘going back to our roots’. Through using the cultural memory of healing and decolonizing healing. From a world view perspective, mental health is increasingly being framed through the decolonial lenses as a culturally embedded, relational concept rather than a purely biomedical condition.

Across indigenous, Global South, and ecological psychology perspectives, well-being, is understood as a balance between the individual, community, land, and spiritual world. This challenges western psychiatric models, that often prioritize diagnosis and symptom reduction and instead emphasizes, cultural memory, relational healing and connection to nature as a central psychological health [17].

Recent ecological frameworks argue that reconnecting people with nature and community systems can function as both healing and epistemic repair, restoring ways of knowing that we are historically marginalized through colonial systems of knowledge production [18,19].

In the Kenyan context, decolonizing mental health is closely tied to the recognition that colonial psychiatry, disrupted indigenous healing systems and community based approaches to distress, replacing them with under resource biomedical models. As a result, many communities continue to rely on culturally grounded and informal systems of care, including family network, spiritual practices and nature-based coping. Emerging legal and policy works in Kenya call for mental health systems that integrate local knowledge, community participation and culturally, responsive frameworks rather than imported models that do not fully reflect lived realities [20].

Within this space, eco-therapy and nature based healing can be seen as a form of epistemic repair, reconnecting individuals to land, environment and community sources of psychological resilience and identity. This aligns with the broader African holistic concepts of well-being where mental health is inseparable from social and ecological harmony [19]. In many African contexts, healing embodiment has always been rooted in movement, rhythm, storytelling, community, and connection to land. Positioning eco-therapy within this context is critical. Rather than framing them as an external or imported practice, it should be understood as complementary to existing indigenous knowledge systems. This alignment strengthens cultural relevance, enhances participation, and reinforces a sense of belonging. It also contributes to a broader project of decolonizing mental health care, ensuring that interventions are not only effective but culturally grounded and meaningful.

Eco-therapy can be conceptualized as a form of epistemic repair because it challenges dominant understandings of mental health that have historically separated human well-being from ecological, relational and embodied experiences. Epistemic repair refers to addressing harms created when certain forms of knowledge are marginalized, silenced or considered less legitimate while other forms of knowledge become dominant [21]. Within modern biomedical and psychological systems, mental health has often been understood primarily through individual symptoms, internal psychological processes and clinical interventions. While these approaches have contributed significantly to understanding and treating mental distress eco-psychology argues that this individualistic orientation can overlook the broader ecological, social and relational contexts within which human suffering emerges [22,23]. From an eco-psychological perspective, the separation of humans from the natural world is not on an environmental issue but also a psychological and cultural one.

Roszak argued that modern societies have developed forms of knowledge that position humans as separate from ecological systems, creating a psychological disconnection that can contribute to feelings of alienation, anxiety and loss of meaning [22]. Eco-therapy responds to this disconnection by restoring attention to relationships between the self, others and the environment. In this sense, nature is not simply used as a tool for relaxation, but it becomes part of a broader understanding of wellbeing in which human health is interconnected with ecological relationships. This represents epistemic repair because eco-therapy expands what is recognizing as legitimate knowledge about healing. Rather than viewing wellbeing only through cognitive or biological frameworks, it incorporates embodied experiences, sensory awareness, place- based knowledge and relational understandings of care [3]. These perspectives align with indigenous and community- based knowledge systems that have long emphasized interconnected relationships between people, land and collective responsibility, although such systems have frequently been marginalized within colonial knowledge structures [23].

Importantly, epistemic repair does not mean replacing scientific knowledge with indigenous knowledge or romanticizing traditional practices; rather, it involves creating dialogue between different knowledge systems and recognizing multiple pathways to understanding human well-being [21]. Within the Kenyan context, this perspective is particularly significant because colonial histories have influenced which forms of knowledge or are prioritized within institutions, including health and education systems. Eco-therapy provides an opportunity to critically reconsider well-being by acknowledging ecological relationships, community practices and embodied experiences alongside psychological science. However, this process must avoid extracting cultural knowledge or presenting indigenous relationships with nature as a lost ideal. Instead, communities should participate as knowledge creators who shape how ecological and cultural understandings of well-being and interpreted and applied [24,25].

Therefore, eco-therapy as epistemic repair is not simply about bringing nature into mental health interventions. It represents a shift in how distress and healing are understood: from an exclusively individual problem toward a relation experience shipped by bodies, communities, histories and environments. By restoring attention to these interconnected dimensions, eco-therapy contributes to developing mental health approaches that are more holistic, culturally responsive and relevant to realities of young people experiencing disconnection within contemporary societies.

## Eco-therapy through the critical lens

Reimagining peer mental health support in this model requires a critical examination of the historical and structural contexts that shape whose knowledge systems are recognized as legitimate. Colonial systems of knowledge production have often privileged western biomedical and individualistic approaches, while marginalizing indigenous and community based understandings of well-being, healing, and relationships with the natural world [25,26].In many African contexts just like in Kenya, Indigenous Knowledge Systems have historically emphasized relation understandings of well-being where individual health is connected to community, environment, spirituality and collective responsibility [27,28].These perspectives challenge dominant models that often position mental health primarily as an individual experience detached from broader social and ecological contexts.

However, recovering marginalized knowledge systems should not be understood as a simple return to a romanticized colonial past or an assumption that historical practices alone provide solutions to contemporary mental health challenges. Such an approach risks overlooking the complexities of past societies and reducing knowledge to static traditions. Instead, de-colonial approaches call for critical engagement with multiple knowledge systems, recognizing the value of indigenous, scientific and emerging forms of knowledge in creating more inclusive futures [21,29].

This involves moving beyond knowledge hierarchies that position one system as superior toward a more integrative approach where diverse perspectives can inform responses to current changes. Within this framework eco-therapy represents not a return to an unchanged past, but a process of knowledge integration and future-making. It combines ecological understanding, psychological research, embodied practices, and culturally grounded perspectives on human- environment relationships to develop approaches that respond to contemporary mental health needs. Research demonstrates that engagement with the natural environment is associated with reduced psychological distress, lower stress levels, and enhanced wellbeing [16,30].

Therefore, this model does not reject modern psychological knowledge nor attempt to recreate a return to the past rather it creates a bridge between different forms of knowledge, integrating evidence with connection, community and natural environment connection. Through the synthesis eco therapy is shown to form part of mental health models that are local, culturally responsive, and globally relevant, recognizing that healing involves individuals, but also relationships with each other and the environment within which people exist. Although eco-therapy and nature based interventions offer promising possibilities for addressing, stress, anxiety and disconnection, they require critical examination to avoid reproducing simplified narratives about nature, culture and healing. One major critique is that some forms of eco-therapy risk romanticizing nature by presenting it as inherently restorative, peaceful and universally healing space while overlooking the social, historical and political realities that shape people’s relationships with environments [31].

Nature is not experienced in the same way by all individuals; access to safe and meaningful natural spaces is influenced by factors such as social economic status, geography, urbanization, disability and histories of land ownership. For example, encouraging a university student experiencing distress to ‘reconnect with nature.’ may assume that they have access to safe green spaces time away from academic and financial pressure and an environment where such practices feel relevant. However, a student experiencing poverty, insecure housing, food insecurity or overwhelming academic demands may experience distress not only because of disconnection from nature but because of structural conditions affecting their well-being.

Similarly, eco-therapy must be cautious of creating a simplistic opposition between nature and modernity where nature is framed as authentic and healing while urban life, technological, capitalism or western psychiatry are portrayed inherently harmful. Such narratives risk reducing complex mental health challenges to a single problem of lost connection with nature while overlooking biological, psychological, social and structural contributors to distress [32]. While biomedical and psychiatric approaches have limitations, they also provide important contributions to understanding and responding to mental health challenges. Therefore, eco-therapy should not be positioned as a replacement for psychiatric care, but as a complementary approach that expands mental health frameworks by recognizing relational, embodied and ecological dimensions of wellbeing.

A further concern relates to the ways indigenous and local ecological knowledge can become eroticized within contemporary wellness movements. There is a risk that indigenous communities are represented as possessing a timeless, natural harmony with the environment, creating an idealized image of ‘traditional wisdom’ that separates these knowledge systems from their historical, political and social contexts [24,33].For instance, a community relationship with land that includes responsibilities, ancestry, collective identity and environmental stewardship may be transformed into a simplified wellness practice focused on your individual relaxation or stress reduction while this occurs, knowledge is extracted from the communities that sustain it and transformed into a resource for external conception. This process risks repeating colonial patterns were marginalized knowledge systems are valued only when they can be incorporated into dominant systems of knowledge production. This issue is particularly important when considering the Kenyan context. Relationships with the land and environment cannot be separated from histories of colonialism, land displacement, economic inequality, urban migration and changing ecological conditions. A nature-based mental health intervention that uses rural landscapes as therapeutic spaces for example, must consider the questions such as: Whose land is being used? Who has access to these spaces? Whose knowledge defines what counts as healing? If communities living within these environments are excluded from decision-making, their landscapes and cultural knowledge may become resources that are consumed without meaningful recognition or benefit.

Therefore, eco-therapy must move beyond viewing communities as sources of cultural practices and instead recognize them as knowledge holders, co-creators and partners and shaping mental health approaches. Community participation is essential in preventing eco-therapy from becoming another extractive practice. Decolonial scholars argue that dominant research and knowledge systems have historically positioned indigenous and local communities as objects of study rather than active producers of knowledge [24]. A more ethical approach requires processes of reciprocity and co-creation where communities contribute to defining well-being, identifying meaningful practices and shaping how interventions are developed and evaluated [25]. For example, communities may contribute knowledge about collective care practices, intergenerational learning, storytelling, relationships with ecosystems and culturally meaningful ways of restoring balance. However, these contributions should not be treated as evidence of a ‘pure’ or untouched connection to nature; they should be understood as evolving knowledge systems that continue to adapt to contemporary realities.

Eco-therapy must also consider the risk of becoming absorbed into capitalist wellness cultures, where practices connected to collective relationships with land and community are transformed into individualized lifestyle choices [34]. In such contexts, wellbeing can be framed as a personal responsibility achieved through mindfulness nature experiences or self-care practices, well larger structural issues such as inequality, academic pressures, environmental injustice and unemployment remain unaddressed. For university students, for example a mindful walk or nature- based activity may support emotional regulation, but it cannot substitute for institutional challenges, financial support, and social protections. Therefore, the value of eco-therapy does not lie in returning to an imagined pre-colonial past or rejecting modern mental health systems. Rather, it’s potential lies in creating new possibilities through dialogue between ecological knowledge, psychological research, community experiences and contemporary realities. A critically informed eco-therapeutic approach ask not only whether nature can support healing, but also who defines healing, whose knowledge is recognized, who has access to this practices and whether this approaches challenge or reproduce existing inequalities. In this essay, eco-therapy is not just looked at from the lens of going back to precolonial periods or just being seated outside in nature; but as a reminder of the reciprocal relationships people share in communities.

## From participants to co-creators of healing

Within an eco-therapy framework, students shift from being passive recipients of mental health support to active co-creators of wellbeing through participatory, peer-led, and nature-based engagement. This occurs when students are involved not only in experiencing guided activities but also in shaping, leading, and sustaining them within their peer communities. In practice, trained peers may organize and facilitate mindful walks, lead outdoor reflection circles, initiate gardening or campus greening projects, participate in conservation or clean-up activities, and co-design simple wellbeing practices within shared campus spaces. Alongside supporting one another emotionally through peer dialogue and shared reflection, students also develop a sense of responsibility toward their physical environment by caring for green spaces, maintaining cleanliness, planting trees, and engaging in sustainability-oriented actions. Through these roles, eco-therapy functions as a collective process of meaning-making where healing is both relational and ecological. This participatory approach strengthens belonging, reduces stigma around help-seeking, burnout. This participatory model strengthens agency, reduces stigma, and fosters belonging. It also shifts students from cycles of cognitive overload into structured moments of embodied restoration. In doing so, it reframes wellbeing as a shared, relational, and ecological process rather than an individual burden as students experience themselves not only as recipients of care but as contributors to both community wellbeing and environmental stewardship.

## The ripple effect of embodied youth led eco-therapy approach

The benefit of eco-therapy is threefold: collective improved mental wellbeing, heightened bodily and emotional awareness and, as a result of deep connection with nature and environmental care. Campus based sustainability studies demonstrate that environmental actions that are made visible, collective and socially reinforced; student engagement increases significantly often resulting in measurable improvements in pro-environmental behavior- [35–37] protecting the same nature where bodily and mental healing comes from. Taken together, these findings suggest the existence of a favorable system in which mental health, embodied well-being practices and environmental care operate through the same underlying mechanism: students are more likely to participate if engagement is sustained and there is a co-creation of systems of care both for mental wellbeing and the environment that are peer led and socially embedded.

This positions universities not only as service providers, but as co-constructed ecosystems of care where students actively shape both well-being and environmental outcomes through integrated peer-led practices. When young people engage in embodied and ecological practices, the impact extends beyond the individual. They become more emotionally aware, more socially connected and environmentally conscious. These changes ripple outward into peer relationships, family systems and institutional culture. Youth led wellness spaces thus become sites for both personal healing and and societal transformation.

A holistic model that recognizes the interdependence between self, society and nature fosters empathy, grounding and shared responsibility for wellbeing qualities essential for both individual healing and collective resilience.

## Conclusion; Rethinking the future of care in equity

In conclusion, eco-therapy offers a more than a collection of nature-based activities; it provides a holistic framework for fostering wellbeing, connection and epistemic repair within university settings and even beyond. By reconnecting students with nature, community and embodied ways of knowing, eco-therapy creates opportunities to address mental health challenges while fostering resilience, belonging and collective care. The integration of peer support further strengthens this approach by promoting mutual learning, empowerment and social connectedness.

Eco-therapy’s significance lies not only in their effectiveness, but in their accessibility. These practices are low cost, scalable and adaptable to peer-led formats making them particularly relevant in resource limited settings like Kenyan Universities. They can be integrated into classrooms, outdoor spaces and informal student gatherings reducing barriers to access while increasing engagement. This positions them as practical complements not replacements to formal mental health systems.

However, universities seeking to implement ecotherapy based peer support initiatives must remain attentive to issues of equity, accessibility and inclusion. Without careful consideration, such interventions risk reproducing existing inequalities by privileging students who have greater access to green spaces, leisure time, cultural familiarity with nature-based practices or fewer physical and financial barriers to participation. Institutions should therefore ensure that programs are co-designed with diverse student communities and adapted to different cultural, social and environmental contexts.

Future research and practice should examine challenges related to institutional support, funding, facilitator training, risk management and the long-term sustainability of ecotherapy programs within higher education. Addressing these considerations will be critical to ensuring that ecotherapy functions not only as a wellbeing intervention but also as a genuinely inclusive and equitable framework for healing, connection and transformation in university communities.
